# Deciphering Biosynthesis Mechanism and Solution Properties of Cyclic Amylopectin

**DOI:** 10.3390/foods13213474

**Published:** 2024-10-30

**Authors:** Ruolan Li, Yaning Shi, Ming Miao

**Affiliations:** 1 State Key Laboratory of Food Science & Technology, Jiangnan University, 1800 Lihu Avenue, Wuxi 214122, China; liruolan5750@163.com; 2 College of Food Science and Technology, Nanjing Agricultural University, Nanjing 210095, China

**Keywords:** starch, branching enzyme, cyclic amylopectin, biosynthesis, solution properties

## Abstract

A novel cyclic amylopectin (CA) was synthesized from waxy corn starch (WCS) using *Bacillus stearothermophilus* branching enzyme (BstBE), providing insights into its biosynthesis mechanism and solution properties. During the first 4 h, BstBE partially cyclized WCS, producing 68.20% CA with a significantly reduced molecular weight (M_W_), from 8.98 × 10⁶ to 3.19 × 10⁴ g/mol and a lower polymer dispersity index (PDI), decreasing from 1.97 to 1.12. This resulted in a uniform CA structure with shorter chain lengths, particularly increasing DP 3–13, especially DP 7–9. Over the subsequent 4–12 h, the PDI slightly increased to 1.18 as the CA content decreased to 50.48%, with an increase in small ring structures (DP 6–12) of CA, suggesting both ring-opening and ring-downsizing due to continued enzyme catalysis. These results propose a two-stage reaction model: initial cyclization followed bybranching and secondary cyclization. CA exhibited excellent solution properties, with BE-4 and BE-12 samples demonstrating high solubility (≥65 g/100 mL), low viscosity (<0.01 Pa·s), and over 90% light transmittance after 14 days at 4 °C, highlighting its broad application potential.

## 1. Introduction

Starch, a vital renewable resource, is widely used across various industries, including food, pharmaceuticals, and chemicals. However, traditional starch has several limitations, such as poor solubility, high viscosity, and limited stability, which restrict its use in high-value-added products [1,2]. To address these challenges, various modification techniques have been developed to enhance the physicochemical properties of starch, meeting the diverse demands of different sectors. Among these techniques, enzymatic modification stands out due to its safety, health benefits, and minimal by-product formation, making it a promising approach for starch enhancement [3,4].

Branching enzymes (BEs) play a crucial role in this process by catalyzing the α-1,6 transglycosylation reaction, a key step in starch modification. During this reaction, α-1,4 linkages are cleaved, and the cleaved segment is transferred to the C-6 hydroxyl group of a glucose residue on the same or another chain, forming an α-1,6 glycosidic bond [5]. This enzymatic action results in two distinct outcomes in starch: branching and cyclization [6]. The former, which increases the degree of branching, has been extensively studied for its impact on the structural and functional properties of modified starch [7,8,9]. In contrast, the cyclization reaction catalyzed by BEs has received comparatively less attention. Although it is known that some strains produce BEs capable of synthesizing cyclic amylopectin (CA) [10,11], the specific mechanisms underlying this cyclization reaction, particularly in the context of amylopectin’s complex structure, remain inadequately understood.

In this study, we investigated the effect of a branching enzyme derived from *Bacillus stearothermophilus* TRBE14 (BstBE) on the microstructure of amylopectin by modifying waxy corn starch (WCS). The primary aim was to elucidate the synthesis mechanism of cyclic amylopectin (CA). Additionally, we analyzed the solution properties of CA to provide a scientific foundation for the development of novel high-performance starch products.

## 2. Materials and Methods

### 2.1. Material

WCS (99% purity, amylopectin content ~96%) was obtained from Gaofeng Biotechnology Co., Ltd. (Suzhou, China). The branching enzyme BstBE (200 U/mL) was expressed in *Escherichia coli*, and enzyme purification was performed according to previously described methods [11]. Ampicillin, isopropyl-β-d-thiogalactoside, and a D-glucose assay kit (GOPOD Format) were purchased from Sangon Biological Co., Ltd. (Shanghai, China). Amylose (Product number: A0512) from potato starch and glucoamylase from *Rhizopus* sp. were sourced from Sigma-Aldrich Chemical Co. (St. Louis, MO, USA). Isoamylase from *Pseudomonas aeruginosa* and β-amylase from *Bacillus cereus* were obtained from Megazyme (Wicklow, Ireland). All other reagent-grade chemicals were supplied by Sinopharm Chemical Reagent Co., Ltd. (Shanghai, China).

### 2.2. Biosynthesis of Cyclic Amylopectin

The preparation of CA was carried out as described in a previous study [11]. WCS (20%, *w*/*v*) was dissolved in 50 mM phosphoric acid buffer (pH 7) and heated at 90 °C for 1 h. The enzyme (300 U/g of starch) was then added, and the reaction was conducted at 65 °C. Samples were collected at 0, 2, 4, 6, and 12 h, then boiled for 20 min. After boiling, the samples were centrifuged (4 °C, 8000× *g*, 20 min) and lyophilized at −80 °C for 48 h. The samples were labeled BE-0, BE-2, BE-4, BE-6, and BE-12, corresponding to the time of collection.

### 2.3. Determination of CA Content

The CA content was calculated according to a modified method from previous studies [12]. The content of reducing ends was determined using the following formula:

$$\mathrm{The}\;\mathrm{content}\;\mathrm{of}\;\mathrm{reducing}\;\mathrm{end}=\mathrm{the}\;\mathrm{content}\;\mathrm{of}\;\mathrm{reducing}\;\mathrm{sugar}\times \frac{M[\mathrm{CA}]}{M[\mathrm{glucosyl}]}$$

The CA content was calculated as follows:

The content of CA =1 − the content of reducing end

where M[CA] and M[glucosyl] represent the molecular weights of CA and glucosyl, respectively. The reducing sugar content was measured using the 3,5-dinitrosalicylic acid (DNS) method [13]. A 200 μL aliquot of a 10% (*w*/*v*) substrate solution was mixed with an equal volume of DNS reagent and boiled in a water bath for 5 min. After adding 400 μL of deionized water, the absorbance was measured at 550 nm.

### 2.4. ^1^H NMR

The relative content of α-1,6 linkages was determined using an AVANCE III 400 MHz digital NMR spectrometer (Bruker Biospin International AG, Fällanden, Switzerland). For measurements, 30 mg of the sample was dissolved in 0.5 mL of D_2_O, and the temperature was set to 60 °C.

### 2.5. Molecular Weight Distribution Analysis

The molecular weight distribution was analyzed using an HPSEC-MALLS-RI system (Wyatt Technology, Goleta, CA, USA), as described in a previous study [14]. The analysis was performed using OH-pak SB-805 and SB-804 columns (Shodex, 8.0 × 300 mm) with 0.1 M NaNO_3_; as the mobile phase at a flow rate of 0.5 mL/min and a column temperature of 40 °C. The refractive index increment (dn/dc) was set at 0.147 mL/g.

### 2.6. Chain Length Analysis

Chain length analysis was conducted using a Dionex DX-600 system with a CarboPac PA1 column, following the elution conditions described in a previous study [15]. Samples (50 mg) were dissolved in 5 mL of 50 mM acetate buffer (pH 4.5) and treated with 150 U of isoamylase at 45 °C for 24 h. The average chain length (CL) was calculated as

CL = ∑ (DP × percentage of signal peaks).

The average external chain length (ECL) was calculated as

ECL = CL × β-amylolysis limit + 2

The average internal chain length (ICL) was derived as

ICL = ECL − 1.

### 2.7. Ring Structure Analysis

The ring structure was analyzed using matrix-assisted laser desorption/ionization time-of-flight mass spectrometry (MALDI-TOF-MS) (Bruker Daltonics Inc., Billerica, MA, USA). The ring structure was isolated following Hiroki Takata’s method [10]. A 10 g sample was dissolved in 100 mL of sodium citrate–phosphate buffer (50 mM, pH 4.5) and incubated with 3000 U of glucoamylase (GA) at 55 °C for 6 h. The reaction was terminated by boiling, and the denatured GA was removed by centrifugation (8000× *g* for 10 min). Ethanol was added in a 10-fold volume to precipitate the product, which was redissolved in sodium citrate–phosphate buffer. This cycle of GA treatment, inactivation, and ethanol precipitation was repeated 14 times until no further glucose was released, as confirmed using a D-glucose assay kit. The resulting structures were labeled as GA-BE-2, GA-BE-4, GA-BE-6, and GA-BE-12. Low-molecular-weight and high-molecular-weight fractions in BE-12 were further separated using a 10 kDa membrane for ring structure analysis.

### 2.8. Solution Properties

#### 2.8.1. Solubility

The solubility was determined according to the method described by Ueda et al. [16]. A 100 mg sample was accurately weighed into a 5 mL centrifuge tube at 25 °C. Deionized water was added dropwise at a rate of 5 μL per drop, with vigorous shaking for 5 min after each addition, until the sample was completely dissolved. The total volume of water added was recorded to determine the sample’s solubility.

#### 2.8.2. Particle Size

Particle size was measured using the Zetasizer Nano ZS (Malvern Instruments Ltd., Malvern, UK) at 25 °C. The refractive index values for the reference and dispersed phases were set at 1.47 and 1.33, respectively.

#### 2.8.3. Viscosity

The viscosity of the sample was measured using a MARS III rheometer (Thermo Haake, Germany) equipped with parallel plates (diameter 40 mm, plate spacing gap 0.1 mm), following the method described by Xie et al. [17]. A 5% (*w*/*v*) sample solution was placed in the measuring element. Angular frequency was varied from 1 to 100 rad·s^−1^, with a constant strain of 1% throughout the scan and a frequency of 0.1 Hz.

#### 2.8.4. Transmittance

The transmittance of a 30% (*w*/*v*) sample solution was monitored daily using a UV-2102PC UV/visible spectrophotometer (Unico Instrument Co., Shanghai, China) according to the method described by You et al. [18]. Photographs were taken to document the solution’s condition. Transmittance (%) was calculated as

Transmittance (%) = 10^−OD620^ × 100.

#### 2.8.5. Freeze–Thaw Stability

A 5% (*w*/*v*) native sample solution was frozen at −20 °C for 24 h, followed by thawing at room temperature (25 °C) for 3 h. This freeze–thaw cycle was repeated three times [19]. Photographs were taken after each cycle to document the solution’s condition.

### 2.9. Statistical Analysis

All experiments were performed in triplicate, and results are presented as mean ± standard deviation. Statistical analysis was conducted using SPSS version 24, with one-way ANOVA applied (*p* < 0.05). Data visualization was carried out using Origin 2022b.

## 3. Results

### 3.1. Biosynthesis of Cyclic Amylopectin

After catalysis with BstBE for varying durations, products with different degrees of modification (BE-0, BE-2, BE-4, BE-6, and BE-12) were obtained, with recovery percentages of 98.67%, 97%, 96.83%, 97.5%, and 98%, respectively. The reducing sugar content slightly increased from 0.37‰ to 4.32‰. Additionally, the relative content of α-1,6 linkages, which corresponds to that of the non-reducing end content, significantly increased over time. BE-12 showed an increase from 5.40% in the control sample (BE-0) to 6.74% (Table 1). These results suggest that BstBE primarily catalyzes transglycosylation with minimal hydrolytic activity [6,11].

As shown in Table 1, the CA content remained consistently between 0 and 1, indicating that BstBE facilitates two types of transglycosylation within amylopectin: cyclization, which produces CA through intramolecular transglycosylation, and branching, which forms non-cyclic products lacking ring structures [10,20,21]. The CA content peaked at 68.20% after 4 h of catalysis but decreased to 50.48% in BE-12. This decline suggests that, after initially promoting CA formation, BstBE gradually opened the ring structures through branching, thereby reducing the overall CA content as the reaction progressed. A similar trend was observed in a previous study [22], where branching enzymes from *Bacillus cereus* were found to reduce ring structures during treatment. These observations underscore the dynamic nature of CA modification during enzymatic catalysis, driven by the interaction between BstBE and the amylopectin substrate.

### 3.2. Structure Analysis of Cyclic Amylopectin

#### 3.2.1. Molecular Weight

**Figure 1:**
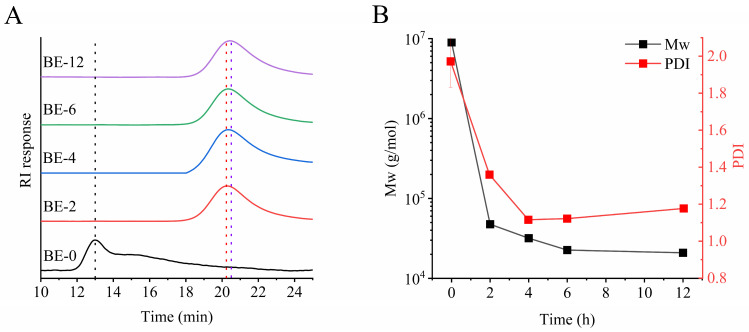
The molecular weight of BstBE-modified amylopectin: (**A**) chromatography profiles; (**B**) Mw and PDI.

The molecular weights of BE-0, BE-2, BE-4, BE-6, and BE-12 were measured (Figure 1). After enzyme catalysis, the elution time peak progressively shifted from 13 min for BE-0 to 20.5 min for BE-12 (Figure 1A), indicating the production of smaller molecular structures. Additionally, the initial broad distribution observed in BE-0 narrowed significantly following BstBE modification, reflecting the formation of more uniform products [12].

As shown in Figure 1B, after 4 h of BstBE modification, the molecular weight (M_W_) rapidly decreased from 8.98 × 10⁶ g/mol for BE-0 to 3.19 × 10⁴ g/mol for BE-4 and continued to decline gradually to 2.09 × 10⁴ g/mol for BE-12. Using the formula $\bar{DP}$_W_ = (M_W_ − 23)/162, the weight-average degree of polymerization ($\bar{DP}$_W_) of BE-4 and BE-12 was calculated to be 196.52 and 128.93, respectively. Based on the cluster structure of amylopectin, the branch chains were classified into A-chains, B1-chains, B2-chains, and B3-chains, corresponding to different chain lengths of DP < 13, DP 25–36, and DP > 36, respectively [23]. These findings suggest that BstBE preferentially cyclizes the B-chains of amylopectin to form CA.

The polydispersity index (PDI) of BE-2, BE-4, BE-6, and BE-12 decreased significantly compared to the control (BE-0), approaching values close to 1, which indicates improved uniformity resulting from enzyme modification. Similarly, Roussel et al. found that the branching enzyme form *Rhodothermus obamensis* produced smaller and more uniform clusters derived from branched or unbranched α-glucans, showing a strong preference for amylopectin over linear fragments as a substrate [21]. Combined with the observed trend in M_W_, these results suggest that BE modification of amylopectin produces smaller, more uniform CA structures.

Unlike the consistent decrease in M_W_, the PDI of BE-4 increased from 1.12 to 1.18 for BE-12. This unexpected increase suggests a more complex behavior that cannot be explained by conventional branching models, highlighting the need for further structural investigation of CA to analyze the underlying mechanisms.

#### 3.2.2. Chain-Length Distribution

**Figure 2:**
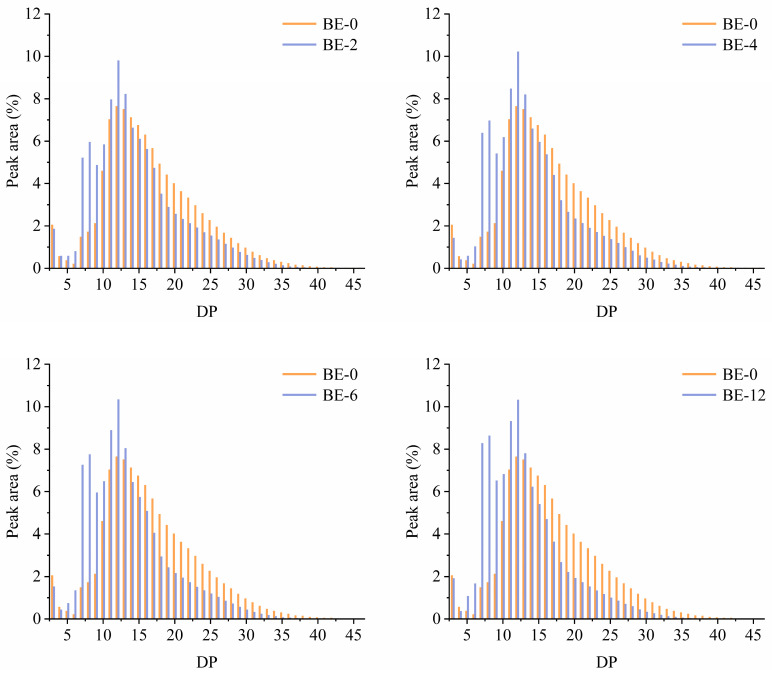
Chain-length distribution of BE-modified amylopectin.

The chain-length distributions for BE-2, BE-4, BE-6, and BE-12, compared to the control (BE-0), are shown in Figure 2. In contrast to BE-0, the modified samples (BE-2, BE-4, BE-6, and BE-12) displayed a significant shift in chain-length distribution, with a decrease in longer segments (DP > 13) and an increase in shorter segments (DP 3–13), particularly in the DP 7–9 range. These findings suggest that BstBE preferentially targets B2- and B3-chains (DP > 13) and selectively combines and transfers fragments with DP 3–13, especially DP 7–9 fragments. These shorter fragments then act as donors for cyclization or branching, resulting in the production of CA with a more compactly branched structure [24]. The substantial increase in chain segments with DP 7–9 may be attributed to cyclization reactions, as previous studies have reported that the average ring span length of BstBE-synthesized ring structures is 5.6 [11].

As shown in Table 1, both CL (average chain length) and ECL (average external chain length) decreased over time, from 16.95 and 12.36 in BE-0 to 13.09 and 8.87 in BE-12, respectively. This indicates that BstBE catalyzes the breakdown of B-chain segments (DP > 13) into shorter chains. In contrast to CL and ECL, the ICL (average internal chain length) increased from 3.18 in BE-2 to 3.35 in BE-4. As reported in previous studies [25,26], this increase occurs because BstBE transfers cleaved sugar chain segments to the non-reducing end of the acceptor chain, thereby extending the distance between branch points and increasing the ICL. Additionally, the formation of ring structures within CA further contributes to this increase, as the ICL in the ring structures corresponds to the length of α-1,4 chains sequentially linked through α-1,6 bonds.

However, with prolonged reaction time, the ICL decreased from 3.35 in BE-4 to 3.23 in BE-12, indicating that BstBE catalyzes the ring-opening during the later stages of the reaction (4–12 h). This shift in ICL dynamics indicates a transition from branching and ring formation to the degradation of ring structures, underscoring the complexity of BstBE-catalyzed modifications over time.

#### 3.2.3. Molecular Mass of the Ring Structures

The molecular mass of the ring structures was determined using matrix-assisted laser desorption/ionization time-of-flight mass spectrometry (MALDI-TOF-MS), renowned for its sensitivity, accuracy, and resolution, making it ideal for identifying cyclic glucans [27]. Multiple peaks ranging from 1000 to 7000 g/mol were observed for each sample, aligning with theoretical values for cyclic rather than non-cyclic glucans (Table A1 and Table A2), as noted in a previous study [28].

**Figure 3:**
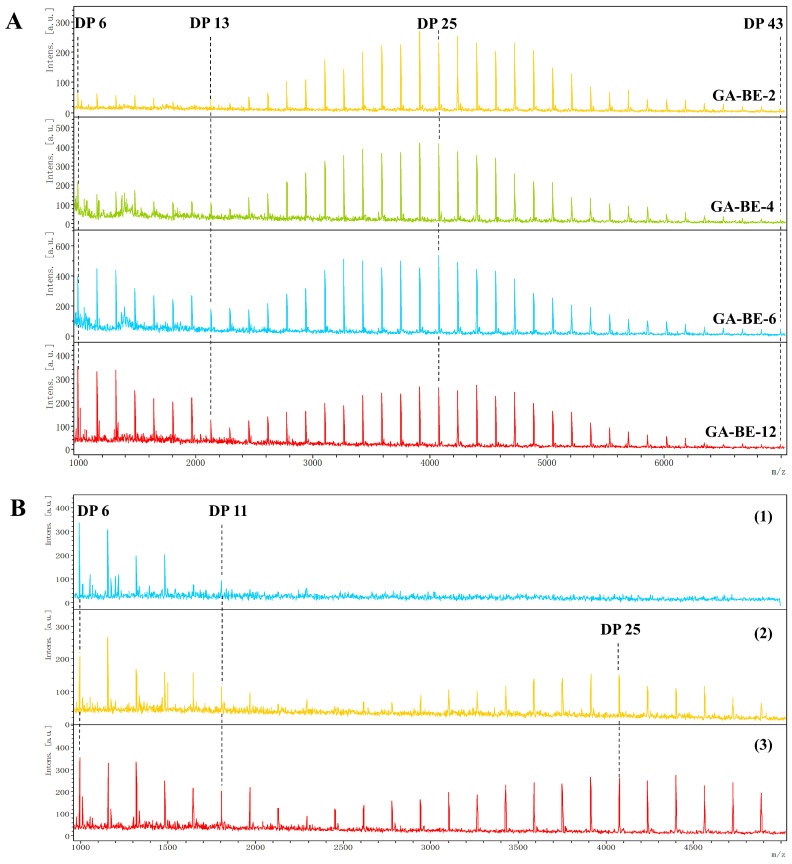
MALDI-TOF-MS mass spectra of ring structures in cyclic amylopectin (**A**); composition of ring structures in BE-12 (**B**): low-molecular-weight molecules of BE-12 (1); ring structures in high-molecular-weight component of BE-12 (2); ring structures of BE-12 (3).

As shown in Figure 3A, BE-2 predominantly contained large ring structures with DP 13–43. Following the BstBE reaction, there was a significant increase in the proportion of small ring structures with DP 6–12, alongside a decrease in large ring structures. This shift in ring size distribution, combined with the trend in CA content (Table 1), indicates that BstBE effectively reduces the amount of large ring structures while increasing the content of smaller rings. The reduction in large ring structures is attributed to the ring-opening effect of BstBE during branching. The increase in smaller rings may result from the secondary cyclization of initially formed large rings or the formation of new free small cyclic glucans through the intrachain cyclization of A-chains.

To further investigate the synthesis of these small ring structures, the ring structure distribution in BE-12 was analyzed (Figure 3B). Mass spectrometry peaks of low-molecular-weight molecules in BE-12 (Figure 3B(1)) corresponded to the theoretical molecular masses of cyclic glucans with DP 6–13. Similarly, Ren et al. identified free cyclic glucans with a DP of 9–12 in BE-modified tapioca starch [26], suggesting that BE catalyzes the intrachain cyclization of amylopectin A-chains to produce free cyclic glucans. The mass spectrum of the ring structures in the high molecular weight component of BE-12 (Figure 3B(2)) revealed peaks corresponding to both small ring structures (DP 6–12) and large ring structures (DP 13–43), consistent with the ring structures observed in BE-12 in Figure 3B(3). These findings suggest that while BE catalyzes the formation of free small cyclic glucans from A-chains, initially formed large rings in CA undergo significant secondary cyclization, converting large rings into smaller ring structures.

### 3.3. Biosynthesis Model of CA

The biosynthesis of cyclic amylopectin (CA) involves a complex interaction between the substrate and BstBE. As described in the structural analysis (Section 3.2), CA formation is characterized by a time-dependent decrease in molecular weight (Mw) and significant changes in CA content, polydispersity index (PDI), internal chain length (ICL), and the molecular distribution of ring structures. These changes reflect the dual role of BstBE in promoting both branching and cyclization of B-chains (DP > 13) throughout the reaction.

#### 3.3.1. Enzymatic Specificity of BstBE

While all branching enzymes (BEs) catalyze branching reactions, BstBE stands out due to its dual catalytic capability, uniquely performing both branching and cyclization. This specificity for cyclization can be attributed to BstBE’s distinctive structural features, particularly its carbohydrate-binding module (CBM48) and active site configuration [29]. Critical residues, such as Lys90 and Glu112, bind substrates via hydrogen bonds, while Trp60 engages in π-stacking interactions with ring structures, facilitating cyclization.

The enzymatic specificity of BstBE includes substrate preference, recognition of substrate chain length, and selection of transfer segments. BstBE demonstrates a strong preference for amylopectin, producing products with a narrow molecular weight distribution [20]. It predominantly recognizes and targets the B-chains of amylopectin (DP > 13), likely due to the short loop structures between CBM48 and domain A, which enhance effective interaction with these long chains [30,31]. For transfer segment selection, BstBE primarily transfers chain fragments of DP 7–9, contributing to A-chain formation through branching and to ring structure formation through cyclization. These unique enzymatic properties make BstBE highly efficient in synthesizing cyclic amylopectin and enhancing its functional characteristics.

#### 3.3.2. Two-Stage Biosynthesis of CA

**Figure 4:**
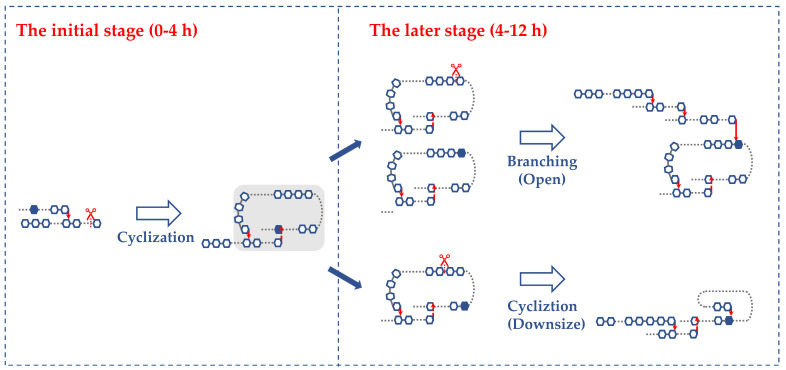
Enzymatic synthesis of cyclic amylopectin: two-stage reaction model.

As illustrated in Figure 4, CA biosynthesis by BstBE occurs in two distinct stages. In the early stage (0–4 h), BstBE primarily targets the B-chains of amylopectin, promoting the formation of large rings (DP 14–43) (Figure 3A), leading to a rapid reduction in molecular weight (Table 1). This stage is accompanied by the branching of B-chains (DP > 13), resulting in an increase in the overall degree of branching and the formation of A-chains, contributing to a more compact structure. In the later stage (4–12 h), the large ring structures serve as substrates for further transglycosylation. BstBE either opens these rings through branching, reducing the CA content, or catalyzes secondary cyclization, converting large rings into smaller ones (DP 6–13). This prolonged catalysis is reflected in the increased PDI of the products.

### 3.4. Solution Properties of CA

**Figure 5:**
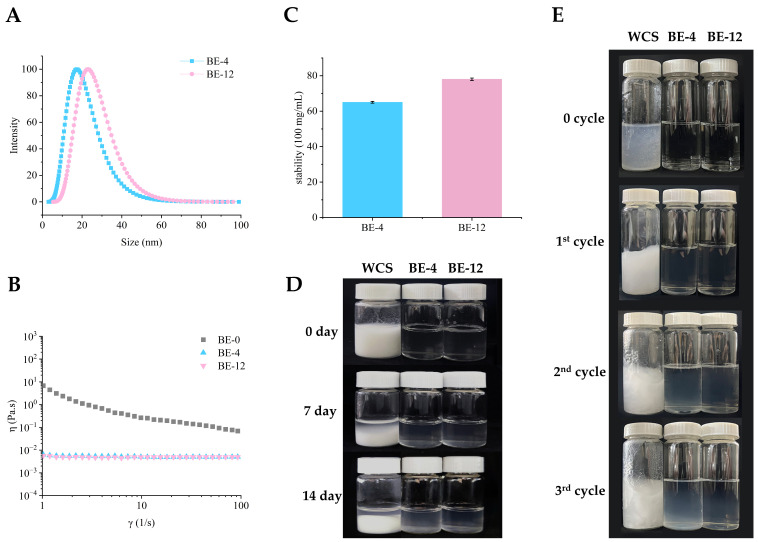
Solution properties of BE-4 and BE-12: (**A**) particle size distributions of BE-4 and BE-12; (**B**) steady shear rheological curves of BE-4 and BE-12; (**C**) solubility of BE-4 and BE-12; (**D**) real-time state diagram of 30% (*w*/*v*) WCS and BE-modified amylopectin solutions stored over time; (**E**) real-time state diagram of 5% (*w*/*v*) gelatinized WCS, BE-4 and BE-12 during freeze–thaw cycles.

The solution properties of BE-4 (the first-stage product) and BE-12 (the second-stage product), including particle size, viscosity, solubility, storage stability, and freeze–thaw stability, are presented in Figure 5.

As shown in Figure 5A, the particle sizes of BE-4 and BE-12 solutions in aqueous media were measured at 17.36 nm and 22.98 nm, respectively. Such small particle sizes enhance Brownian motion, which is crucial for maintaining suspension stability and preventing sedimentation [8]. Figure 5B shows that the viscosity of a 5% (*w*/*v*) CA solution is notably low (<0.01 Pa·s), significantly lower than that of traditional starches (BE-0). In Figure 5C, CA exhibits exceptional solubility, with BE-4 and BE-12 reaching values of 65.00 g/100 mL and 78.00 g/100 mL, respectively. This high solubility is due to CA’s highly branched structure, which facilitates extensive hydrogen bonding with water molecules [32]. These results suggest that biosynthesized CA is a typical low-viscosity, highly water-soluble nanomaterial, making it suitable for applications requiring clear and stable solutions.

**Figure A1:**
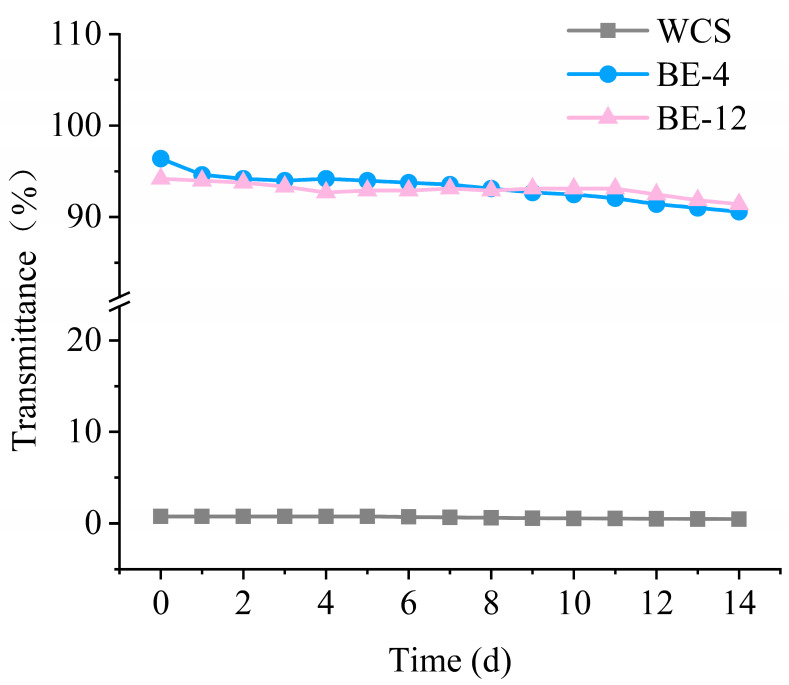
Transmittance of 30% (*w*/*v*) solution of BE-4 and BE-12 stored at 4 °C for 14 days.

As shown in Figure 5D, a 30% (*w*/*v*) solution of BE-4 and BE-12 maintained over 90% light transmittance (Figure A1) and exhibited no sedimentation after 14 days at 4 °C, indicating excellent storage stability. Similarly, You et al. observed that branching enzyme-modified maltodextrin significantly improved solution stability [18]. Additionally, Figure 5E illustrates that, while a 5% (*w*/*v*) gelatinized WCS solution forms a porous structure after three freeze–thaw cycles, 5% (*w*/*v*) BE-4 and BE-12 solutions showed minimal sedimentation, with BE-12 exhibiting the highest stability. This result indicates that the freeze–thaw stability of CA solutions is superior to that of traditional starches. Ma et al. also reported that incorporating 2% (*w*/*v*) of BE-modified products significantly increased the storage modulus and improved the freeze–thaw stability of carrageenan gels [33].

Overall, the unique combination of low particle size, low viscosity, high solubility, and excellent stability underscores the potential of CA for various industrial applications [34]. In the food and beverage sector, CA’s high solubility and low viscosity make it ideal for clear and stable solutions in beverages and sauces [35], where conventional starches often lead to undesirable cloudiness or thickening. Its resistance to retrogradation and aging is particularly advantageous for baked goods and processed foods, helping to extend shelf life and maintain quality [19,36]. In the pharmaceutical field, CA’s small particle size, stability, and unique structural features make it a potential carrier in drug delivery systems [11,37]. These characteristics highlight CA’s versatility and potential for diverse applications, warranting further exploration and development.

## 4. Conclusions

The results of this study demonstrate that the BstBE effectively synthesizes a novel type of CA from WCS. Initially, BstBE catalyzed both the cyclization and branching of B-chains in amylopectin, leading to the formation of CA with large ring structures, highly branching characteristics, and a narrow size distribution. As the reaction progressed, BstBE further transglycosided these large rings, either converting them into linear chains or forming smaller rings. Compared to native starch, CA exhibited a lower molecular weight, a higher degree of branching, shorter branch chains, and a hydrophobic annular cavity at its core, which significantly enhances its solubility, solution stability, and freeze–thaw stability. These findings provide valuable insights into the biosynthesis mechanism of CA and highlight its potential applications as a stabilizer in functional beverages, where it can improve shelf life and product consistency. Furthermore, the hydrophobic annular cavity of CA positions it as an effective nanocarrier for the delivery of bioactive compounds or drugs, making it highly applicable in the pharmaceutical and food industries.

## Figures and Tables

**Table 1:** Structural parameters of BE-modified amylopectin.

Sample	Reducing Sugar (‰)	CA (%)	R_α-1,6_ (%)	CL	ECL	ICL
BE-0	0.37 ± 0.02 ^e^	0.00 ± 0.00 ^e^	5.40 ± 0.02 ^d^	16.95 ± 0.31 ^a^	12.36 ± 0.19 ^a^	3.58 ± 0.19 ^a^
BE-2	2.20 ± 0.02 ^c^	43.34 ± 0.58 ^d^	5.89 ± 0.01 ^c^	14.63 ± 0.19 ^b^	10.54 ± 0.11 ^b^	3.18 ± 0.11 ^b^
BE-4	1.85 ± 0.02 ^d^	68.20 ± 0.37 ^a^	5.99 ± 0.02 ^c^	14.09 ± 0.09 ^c^	9.77 ± 0.05 ^c^	3.35 ± 0.05 ^ab^
BE-6	3.53 ± 0.03 ^b^	56.87 ± 0.31 ^b^	6.20 ± 0.04 ^b^	13.64 ± 0.13 ^c^	9.36 ± 0.07 ^d^	3.28 ± 0.07 ^b^
BE-12	4.32 ± 0.03 ^a^	50.48 ± 0.39 ^c^	6.74 ± 0.07 ^a^	13.09 ± 0.19 ^d^	8.87 ± 0.07 ^e^	3.23 ± 0.07 ^b^

Rα-1,6, ratio of α-1,6 linkages; CL, average chain length; ECL, average external chain length; ICL, average internal chain length. Different letters within the same column indicate statistical differences (*p* < 0.05).

## Data Availability

The data presented in this study are available on request from the corresponding author (The authors do not have permission to share data).

## Appendix A

**Table A1:** Molecular mass of ring structures in cyclic amylopectin.

DP_W_	Theoretical Molecular Mass (g/mol)		Experimental Molecular Mass (g/mol)			
	Non-Cyclic Glucan	Cyclic Glucan	GA-BE-2	GA-BE-4	GA-BE-6	GA-BE-12
6	1013	995	995.29	995.37	995.29	995.29
7	1175	1157	1157.35	1157.41	1157.31	1157.35
8	1337	1319	1319.41	1319.49	1319.40	1319.40
9	1499	1481	1481.46	1481.54	1481.44	1481.49
10	1661	1643	1643.51	1643.63	1643.48	1643.52
11	1823	1805	1805.59	1805.66	1806.55	1805.53
12	1985	1967	1967.54	1968.74	1967.62	1967.63
13	2147	2129	2129.69	2129.76	2129.65	2129.71
14	2309	2291	2291.73	2291.85	2292.75	2291.81
15	2471	2453	2454.81	2454.89	2454.76	2454.89
16	2633	2615	2615.83	2615.92	2615.82	2616.95
17	2795	2777	2777.91	2778.99	2777.82	2777.96
18	2957	2939	2939.95	2942.04	2939.88	2939.89
19	3119	3101	3101.98	3103.09	3101.91	3102.01
20	3281	3263	3264.03	3265.12	3263.93	3265.09
21	3443	3425	3426.07	3427.18	3425.97	3427.10
22	3605	3587	3588.11	3589.20	3588.02	3588.16
23	3767	3749	3750.14	3752.22	3751.09	3750.18
24	3929	3911	3912.19	3913.27	3912.09	3912.23
25	4091	4073	4074.20	4075.31	4076.11	4074.26
26	4253	4235	4236.23	4237.34	4238.13	4236.29
27	4415	4397	4398.27	4399.37	4398.15	4398.30
28	4577	4559	4560.28	4562.37	4560.17	4560.34
29	4739	4721	4722.29	4724.39	4722.21	4722.35
30	4901	4883	4884.30	4886.42	4884.21	4884.34
31	5063	5045	5046.30	5048.46	5048.27	5046.39
32	5225	5207	5207.30	5209.41	5208.27	5208.37
33	5387	5369	5370.34	5372.42	5369.27	5372.32
34	5549	5531	5532.33	5534.36	5532.26	5535.44
35	5711	5693	5693.33	5696.39	5694.23	5695.40
36	5873	5855	5856.31	5859.38	5855.20	5859.38
37	6035	6017	6018.31	6020.53	6019.28	6020.56
38	6197	6179	6180.24	6183.38	6181.14	6180.26
39	6359	6341	6343.31	6345.34	6346.43	6344.62
40	6521	6503	6505.31	6506.39	6505.84	6506.05
41	6683	6665	6667.17	6668.66	6666.88	6667.95
42	6845	6827	6829.11	6830.44	6830.00	6830.16
43	7007	6989	6992.48	6993.52	6991.49	6992.86

Note: The ring structures that are resistant to GA are labeled as GA-BE-2, GA-BE-4, GA-BE-6, and GA-BE-12.

**Table A2:** Molecular mass of ring structures in different components in BE-12.

DP_W_	Theoretical Molecular Mass (g/mol)		Experimental Molecular Mass (g/mol)		
	Non-Cyclic Glucan	Cyclic Glucan	BE-12 (1)	GA-BE-12 (2)	GA-BE-12
6	1013	995	995.31	995.14	995.32
7	1175	1157	1157.36	1157.19	1157.37
8	1337	1319	1319.38	1319.23	1319.43
9	1499	1481	1481.44	1481.31	1481.48
10	1643	1661	1643.54	1643.30	1643.53
11	1823	1805	1805.53	1805.36	1805.59
12	1985	1967	1967.53	1967.45	1967.62
13	2147	2129	-	2129.42	2129.68
14	2309	2291	-	2291.53	2291.74
15	2471	2453	-	2453.51	2453.76
16	2633	2615	-	2616.62	2615.83
17	2795	2777	-	2778.64	2777.88
18	2957	2939	-	2941.74	2939.92
19	3119	3101	-	3102.89	3101.95
20	3281	3263	-	3264.92	3264.00
21	3443	3425	-	3426.05	3426.04
22	3605	3587	-	3588.12	3588.07
23	3767	3749	-	3750.20	3750.10
24	3929	3911	-	3912.26	3912.12
25	4091	4073	-	4074.40	4074.16
26	4253	4235	-	4236.49	4236.17
27	4415	4397	-	4398.58	4398.19
28	4577	4559	-	4560.68	4560.23
29	4739	4721	-	4724.77	4722.26
30	4901	4883	-	4886.90	4884.27

Note: The ring structures that are resistant to GA are labeled as GA-BE-12. low-molecular-weight molecules of BE-12 are labeled as BE-12 (1); ring structures in high-molecular-weight component of BE-12 are labeled as GA-BE-12 (2); ring structures of BE-12 are labeled as GA-BE-12.

## References

[1] Lu K., Miao M., Ye F., Cui S.W., Li X., Jiang B. (2016). Impact of dual-enzyme treatment on the octenylsuccinic anhydride esterification of soluble starch nanoparticle. Carbohydr. Polym..

[2] Jung S.-J., Song Y.-B., Park C.-S., Yoo S.-H., Kim H.-S., Seo D.-H., Lee B.-H. (2022). Different physicochemical properties of entirely α-glucan-coated starch from various botanical sources. Food Sci. Biotechnol..

[3] Punia Bangar S., Ashogbon A.O., Singh A., Chaudhary V., Whiteside W.S. (2022). Enzymatic modification of starch: A green approach for starch applications. Carbohydr. Polym..

[4] He M., Jiang H., Kong H., Li C., Gu Z., Ban X., Li Z. (2023). Engineering starch by enzymatic structure design for versatile applications in food industries: A critical review. Syst. Microbiol. Biomanuf..

[5] Park I., Park M., Yoon N., Cha J. (2019). Comparison of the Structural Properties and Nutritional Fraction of Corn Starch Treated with Thermophilic GH13 and GH57 alpha-Glucan Branching Enzymes. Foods.

[6] Takata H., Takaha T., Okada S., Takagi M., Imanaka T. (1996). Cyclization Reaction Catalyzed by Branching Enzyme. J. Bacteriol..

[7] Xia C., Zhong L., Wang J., Zhang L., Chen X., Ji H., Ma S., Dong W., Ye X., Huang Y. (2021). Structural and digestion properties of potato starch modified using an efficient starch branching enzyme AqGBE. Int. J. Biol. Macromol..

[8] Kageyama A., Yanase M., Yuguchi Y. (2019). Structural characterization of enzymatically synthesized glucan dendrimers. Carbohydr. Polym..

[9] Chen Y.M., Hu X.T., Lu K.Y., Zhang T., Miao M. (2023). Biosynthesis of maltodextrin-derived glucan dendrimer using microbial branching enzyme. Food Chem..

[10] Takata H., Takaha T., Okada S., Hizukuri S., Takagi M., Imanaka T. (1996). Structure of the cyclic glucan produced from amylopectin by *Bacillus stearothermophilus* branching enzyme. Carbohydr. Res..

[11] Chen C., Lu K.Y., Hu X.T., Liu Y., Cui S.W., Miao M. (2020). Biofabrication, structure and characterization of an amylopectin-based cyclic glucan. Food Funct..

[12] Takata H., Takaha T., Nakamura H., Fujii K., Okada S., Takagi M., Imanaka T. (1997). Production and some properties of a dextrin with a narrow size distribution by the cyclization reaction of branching enzyme. J. Ferment. Bioeng..

[13] Gusakov A.V., Kondratyeva E.G., Sinitsyn A.P. (2011). Comparison of Two Methods for Assaying Reducing Sugars in the Determination of Carbohydrase Activities. Int. J. Anal. Chem..

[14] Feng W., Wang Z., Campanella O.H., Zhang T., Miao M. (2023). Fabrication of phytoglycogen-derived core-shell nanoparticles: Structure and characterizations. Food Chem..

[15] Zhang X., Xu W., Ni D., Zhang W., Guang C., Mu W. (2023). Successful Manipulation of the Product Spectrum of the Erwinia amylovora Levansucrase by Modifying the Residues around loop1, Loop 3, and Loop 4. J. Agric. Food Chem..

[16] Ueda H., Wakisaka M., Nagase H., Takaha T., Okada S. (2002). Physicochemical Properties of Large-Ring Cyclodextrins (CD 18∼CD 21. J. Incl. Phenom. Macrocycl. Chem..

[17] Xie H., Ying R., Huang M. (2022). Effect of arabinoxylans with different molecular weights on the gelling properties of wheat starch. Int. J. Biol. Macromol..

[18] You Y.X., Li Y., Tao J., Li C.M., Gu Z.B., Ban X.F., Kong H.C., Xia H.Y., Tong Y., Li Z.F. (2023). Remarkable improvement in the storage stability of maltodextrin through 1,4-α-glucan branching enzyme modification. Food Hydrocoll..

[19] Gao K., Liu Y., Liu T., Song X., Ruan R., Feng S., Wang X., Cui X. (2022). OSA improved the stability and applicability of emulsions prepared with enzymatically hydrolyzed pomelo peel insoluble fiber. Food Hydrocoll..

[20] Roussel X., Lancelon-Pin C., Viksø-Nielsen A., Rolland-Sabaté A., Grimaud F., Potocki-Véronèse G., Buléon A., Putaux J.-L., D’Hulst C. (2013). Characterization of substrate and product specificity of the purified recombinant glycogen branching enzyme of Rhodothermus obamensis. Biochim. Biophys. Acta BBA Gen. Subj..

[21] Gaenssle A.L.O., Bax H.H.M., Jurak E. (2021). GH13 Glycogen branching enzymes can adapt the substrate chain length towards their preferences via α-1, 4-transglycosylation. Enzym. Microb. Technol..

[22] Takata H., Kato T., Takagi M., Imanaka T. (2005). Cyclization Reaction Catalyzed by *Bacillus cereus* Branching Enzyme, and the Structure of Cyclic Glucan Produced by the Enzyme from Amylose. J. Appl. Glycosci..

[23] Nakamura Y., Kainuma K. (2022). On the cluster structure of amylopectin. Plant Mol. Biol..

[24] Kim E.J., Ryu S.I., Bae H.A., Huong N.T., Lee S.B. (2008). Biochemical characterisation of a glycogen branching enzyme from Streptococcus mutans: Enzymatic modification of starch. Food Chem..

[25] Li C., Wu A.C., Go R.M., Malouf J., Turner M.S., Malde A.K., Mark A.E., Gilbert R.G. (2015). The characterization of modified starch branching enzymes: Toward the control of starch chain-length distributions. PLoS ONE.

[26] Ren J., Li C., Gu Z., Cheng L., Hong Y., Li Z. (2018). Digestion rate of tapioca starch was lowed through molecular rearrangement catalyzed by 1,4-α-glucan branching enzyme. Food Hydrocoll..

[27] Choma A., Komaniecka I. (2011). Characterization of cyclic β-glucans of Bradyrhizobium by MALDI-TOF mass spectrometry. Carbohydr. Res..

[28] Terada Y., Yanase M., Takata H., Takaha T., Okada S. (1997). Cyclodextrins Are Not the Major Cyclic α-1,4-Glucans Produced by the Initial Action of Cyclodextrin Glucanotransferase on Amylose. J. Biol. Chem..

[29] Takata H., Takaha T., Kuriki T., Okada S., Takagi M., Imanaka T. (1994). Properties and active center of the thermostable branching enzyme from Bacillus stearothermophilus. Appl. Environ. Microbiol..

[30] Jiang H., Xie X., Ban X., Gu Z., Cheng L., Hong Y., Li C., Li Z. (2021). Flexible Loop in Carbohydrate-Binding Module 48 Allosterically Modulates Substrate Binding of the 1,4-α-Glucan Branching Enzyme. J. Agric. Food Chem..

[31] Suzuki E., Suzuki R. (2016). Distribution of glucan-branching enzymes among prokaryotes. Cell. Mol. Life Sci..

[32] Feng W., Wang Z., Zhang T., Campanella O.H., Miao M. (2023). Biomimetic synthesis of maltodextrin-derived dendritic nanoparticle and its structural characterizations. Carbohydr. Polym..

[33] Ma W.T., Yuan C., Cui B., Gao T., Guo L., Yu B., Zhao M., Zou F.X. (2024). Highly-branched cyclic dextrin for improvement in mechanical properties and freeze-thaw stability of x-carrageenan gels. Food Hydrocoll..

[34] Wu K., Li C., Li Z., Li Z., Gu Z., Ban X., Hong Y., Cheng L., Kong H. (2024). Highly-branched modification of starch: An enzymatic approach to regulating its properties. Food Hydrocoll..

[35] Takata H., Akiyama T., Kajiura H., Kakutani R., Furuyashiki T., Tomioka E., Kojima I., Kuriki T. (2010). Application of branching enzyme in starch processing. Biocatal. Biotransform..

[36] Li W., Li C., Gu Z., Qiu Y., Cheng L., Hong Y., Li Z. (2016). Relationship between structure and retrogradation properties of corn starch treated with 1,4-α-glucan branching enzyme. Food Hydrocoll..

[37] Kaneo Y., Taguchi K., Tanaka T., Yamamoto S. (2014). Nanoparticles of hydrophobized cluster dextrin as biodegradable drug carriers: Solubilization and encapsulation of amphotericin B. J. Drug Deliv. Sci. Technol..

